# DNA Methylation Regulates a Set of Long Non-Coding RNAs Compromising Hepatic Identity during Hepatocarcinogenesis

**DOI:** 10.3390/cancers14092048

**Published:** 2022-04-19

**Authors:** Miriam Recalde, María Gárate-Rascón, José María Herranz, María Elizalde, María Azkona, Juan P. Unfried, Loreto Boix, María Reig, Bruno Sangro, Maite G. Fernández-Barrena, Puri Fortes, Matías A. Ávila, Carmen Berasain, María Arechederra

**Affiliations:** 1 Program of Hepatology, Centre of Applied Medical Research (CIMA), University of Navarra, 31008 Pamplona, Spain; mrecaldedom@alumni.unav.es (M.R.); mgarate.3@alumni.unav.es (M.G.-R.); jherranz.1@alumni.unav.es (J.M.H.); melizaldea@unav.es (M.E.); mazcona@unav.es (M.A.); magarfer@unav.es (M.G.F.-B.); maavila@unav.es (M.A.Á.); 2 National Institute for the Study of Liver and Gastrointestinal Diseases (CIBERehd, Carlos III Health Institute), 28029 Madrid, Spain; lboix@clinic.cat (L.B.); mreig1@clinic.cat (M.R.); bsangro@unav.es (B.S.); pfortes@unav.es (P.F.); 3 Department of Gene Therapy and Regulation of Gene Expression, Center for Applied Medical Research (CIMA), University of Navarra, 31008 Pamplona, Spain; junfried@unav.es; 4 Barcelona Clinic Liver Cancer (BCLC) Group, Liver Unit, Hospital Clínic de Barcelona, IDIBAPS, University of Barcelona, 08036 Barcelona, Spain; 5 Hepatology Unit, Navarra University Clinic, 31008 Pamplona, Spain; 6 IdiSNA, Navarra Institute for Health Research, 31008 Pamplona, Spain

**Keywords:** lncRNAs, epigenetics, DNA methylation, hepatocellular differentiation, hepatocellular carcinoma, prognosis

## Abstract

**Simple Summary:**

Hepatocarcinogenesis is a long process which implies the loss of hepatic functions. Our effort is to understand the mechanisms implicated in this pathological process in order to contribute to the development of new diagnostic markers and therapeutic targets. In this study we have identified a set of lncRNAs significantly downregulated in hepatocellular carcinoma (HCC) in correlation with the grade of tumor dedifferentiation and patients’ worse prognosis. Mechanistically, our results show that they are related with hepatic differentiation and at least a subset of those lncRNAs are essential to ensure the expression of other hepato-specific genes required for liver function. Moreover, we demonstrate that the expression of these lncRNAs in HCC is silenced by DNA methylation. All in all, we uncover connected epigenetic alterations involved in the progression of liver cancer and identify potential new biomarkers.

**Abstract:**

Background: Long noncoding RNAs (lncRNAs) are emerging as key players in cancer, including hepatocellular carcinoma (HCC). Here we identify the mechanism implicated in the HCC inhibition of a set of lncRNAs, and their contribution to the process of hepatocarcinogenesis. Methods and Results: The top-ranked 35 lncRNAs downregulated in HCC (Top35 LNDH) were validated in several human HCC cohorts. We demonstrate that their inhibition is associated with promoter hypermethylation in HCC compared to control tissue, and in HCC human cell lines compared to primary hepatocytes. Moreover, demethylating treatment of HCC human cell lines induced the expression of these lncRNAs. The Top35 LNDH were preferentially expressed in the adult healthy liver compared to other tissues and fetal liver and were induced in well-differentiated HepaRG cells. Remarkably, their knockdown compromised the expression of other hepato-specific genes. Finally, the expression of the Top35 LNDH positively correlates with the grade of tumor differentiation and, more importantly, with a better patient prognosis. Conclusions: Our results demonstrate that the selected Top35 LNDH are not only part of the genes that compose the hepatic differentiated signature but participate in its establishment. Moreover, their downregulation through DNA methylation occurs during the process of hepatocarcinogenesis compromising hepatocellular differentiation and HCC patients’ prognosis.

## 1. Introduction

The development and progression of hepatocellular carcinoma (HCC) is a complex, multistep process in which the underlying hepatic insufficiency is not only related to the hepatocellular loss but also to the dedifferentiation of the remaining liver parenchyma [1]. It has been clearly demonstrated that the loss of liver differentiation eases HCC development [1] and dictates the prognosis of HCC patients. Moreover, several clinical reports have highlighted that those patients with poorly differentiated or undifferentiated HCCs have a worse prognosis than patients with well-differentiated HCCs [2,3,4].

The specific phenotype displayed by a fully differentiated cell is the result of the expression of a broad but unique combination of genes which determine its identity and thus its function [5]. Multiple mechanisms govern gene expression in an exquisite temporal and cell-type specific manner. Among them, epigenetic mechanisms determine chromatin structure and accessibility, therefore defining gene activity state. Epigenetic regulation of chromatin includes DNA methylation, post-translational histone modifications, nucleosome remodeling and non-coding RNAs (ncRNAs) [6]. The correct expression of a plethora of epigenetic proteins (writer, readers and erasers) and ncRNAs will determine the epigenomic landscape and, thus, the transcriptomic identity of a cell [7]. Consequently, dysregulation of these epigenetic mechanisms compromises the maintenance of cell differentiation and leads to human disorders including cancer [8,9,10].

In this line, we have previously identified a list of long non-coding RNAs (lncRNAs) deregulated in different types of tumors, including HCC [11]. In the present work, we have focused on the 35 top-ranked lncRNAs identified as downregulated in HCC and validated their deregulation in independent human HCC cohorts. Mechanistically, we demonstrate that the expression of these epigenetic players is in turn regulated by another epigenetic mechanism, DNA methylation. We show that promoter DNA hypermethylation is responsible for the downregulation of this set of lncRNAs in HCC. Interestingly, we also demonstrate that this set of lncRNAs is preferentially expressed in the adult healthy liver being not only part of the genes that compose the hepatic differentiated signature, but essential for ensuring the transcription of other hepatic-specific genes. Accordingly, we show that the level of expression of this set of lncRNAs in HCC patients positively correlates with the grade of liver differentiation and patient prognosis. Therefore, our results strengthen the link between epigenetic mechanisms such as DNA methylation and lncRNA expression with liver differentiation, and altogether demonstrate that hepatocarcinogenesis is associated with the DNA methylation mediated downregulation of a set of lncRNAs essential to ensure hepatic differentiation and function.

## 2. Materials and Methods

### 2.1. Human Samples

The study was approved by the Human Research Review Committee of the University of Navarra (CEI 47/2015). Liver samples were provided by the Biobank of the University of Navarra (ISCIII Ref B.0000612) and the Clinic Hospital in Barcelona (BCL-CUN cohort [11]) and were processed following standard operating procedures approved by the Ethical and Scientific Committees. Samples from cirrhotic livers (n = 12) or HCCs (cancer and peritumoral samples; n = 19) were from individuals undergoing partial hepatectomy or liver transplantation. Healthy liver tissues (n = 6) were obtained from individuals with normal or minimal changes in the liver. Informed consent was obtained from each patient and the study protocol conformed to the ethical guidelines of the 1975 Declaration of Helsinki. Clinical data of patients with HCC from The Cancer Genome Atlas (TCGA) database (LIHC cohort) were downloaded from the TCGA database.

### 2.2. Public Datasets

For this study we have analyzed the top 35 lncRNAs downregulated in HCC (Top35 LNDH) identified by Unfried et al. [11]. These differentially expressed lncRNAs were identified by comparison of RNA-seq data from tumor tissues and peritumoral samples from the TCGA database (http://cancergenome.nih.gov/; accessed on 1 October 2017) using a sampling-based strategy (10). Briefly, the matrix of raw counts obtained using the STAR aligner with hg38 assembly and annotated with Gencode version 22 was downloaded from the Genomic Data Commons (GDC) Portal (https://portal.gdc.cancer.gov/; accessed on 1 October 2017). Differential expression analysis was performed with the limma package workflow for RNA-seq data analysis from R/Bioconductor [12] using data from all the 50 peritumoral liver hepatocellular carcinoma (LIHC) samples and 50 tumor samples taken randomly from all the 374 LIHCs as input, and the study was performed 200 times with different sets of 50 tumor samples each time. The set of lncRNAs differentially expressed in all the iterations using the criteria of FDR < 1% was retained and the Top35 LNDH are the object of this work.

The expression of the Top35 LNDH was analyzed in RNASeq data from our own cohort of HCC patients [13], in two additional HCC cohorts (GSE144269 and GSE101432), in fetal and adult liver samples (GSE111845) and in normal tissues (GTEx). A custom bash script was used to ensure the correct and complete download of all RNAseq datasets from Sequence Read Archive (SRA) with an SRAtoolkit (2.9.6-1-centos_linux64) from the National Center for Biotechnology Information (NCBI). Trimgalore version 0.6.0 with Cutadapt version 1.18 was used for reads trimming and quality filtering, and all reads below 20nt were filtered. The mapping step was carried out using STAR version 020201 [14] over genome version hg38. Read counting was performed using HTseq version 0.11.0 [15] and normalized using EdgeR version 3.28.1 [16] in R version 3.6.3 (https://www.R-project.org/; accessed on 1 September 2021). Trimmed mean of M-values (TMM) was selected as the method for normalization.

Public methylome data generated on the Infinium HumanMethylation450 BeadChip (HM450K) TCGA data platform [17] from liver (LIHC), lung (LUSC and LUAD) and breast (BRCA) cancer tissues and matched peritumoral samples from the TCGA database (http://cancergenome.nih.gov/; accessed on 1 December 2021) as well as from primary hepatocytes and HCC cell lines (FLNEO, H801, HCO2, Hep3B, Huh75, LH86, SNU423, SNU449 and HepG2) (GSE60753 and GSE42490) were used to determine the methylation status (β-value) of the CpGs located in the promoter region of 19 out of the 35 lncRNAs. 5000pb upstream from the transcriptional start site (TSS) was used for a relatively comprehensive range of lncRNA promoters. β-values were calculated as the ratio of methylated signal to the sum of the methylated and unmethylated signals. The range of β-values is from 0 (unmethylated) to 1 (completely methylated).

As described above, the clinical data of patients with HCC from TCGA (LIHC cohort) were downloaded from the TCGA database.

### 2.3. Cell Lines Culture and Transfection

Human HCC cell lines PLC/PRF/5 and HepG2 were obtained from the ATCC and were grown in DMEM (Gibco-Life Technology, Madrid, Spain; 41966-029), supplemented with 10% fetal bovine serum (FBS), glutamine and antibiotics. Human non-small cell lung cancer cell line H358 was obtained from the ATCC and grown in RPMI (Gibco-Life Technology; 61870-010) and supplemented with 10% FBS, glutamine and antibiotics. Hepatoma cell line HepaRG was obtained from BioPredic (Rennes, France) and was grown in Williams’ Medium E (Gibco-Life Technology, Madrid, Spain; 2551-022) supplemented with 7.5% FBS, insulin, hydrocortisone and antibiotics. We followed a well-established protocol for in vitro differentiation of progenitor HepaRG cells toward hepatocyte-like cells [18]. Cells were grown at 37 °C in a humidified atmosphere containing 5% CO_2_. Where indicated, cells were exposed to 10 µM of 5-Aza-2′-deoxycytidine (DAC; Sigma, St. Louis, MO, USA; A3656) for three or seven days before harvesting. Individual antisense LNA-GapmeRs (Qiagen) to knockdown the expression of *LINC00844*, *LINC00885*, *FAM99A* and *FAM99B* were designed and used in combination at 50 nM each. As a negative control, antisense LNA-GapmeR-5′FAM provided by Qiagen was used. Cells were transfected using Lipofectamine 2000 (Invitrogene; 11668027) following the manufacturer´s instructions. The sequence of LNA-GapmeRs will be provided upon request.

### 2.4. Total DNA Isolation

Total DNA from frozen tissues and cultured cells was isolated using the Maxwell^®^ RSC Cultured Cells DNA Purification Kit with a Maxwell^®^ RSC 48 instrument (Promega, Madison, WI, USA; AS1620). DNA purity and concentration were measured using a NanoDrop spectrophotometer (Thermo Fisher Scientific, Waltham, MA, USA).

### 2.5. Targeted Bisulfite Sequencing

Genomic DNA from HCC and paired peritumoral tissues (1 µg; n = 7) as well as from PLC/PRF/5 control and treated with 10 µM 5-Aza-2′-deoxycytidine for 7 days (500 ng; n = 3) were used to asses CpG methylation levels in the promoter region of the 8 selected lncRNAs by bisulfite sequencing (Zymo Research Corporation, Irvine, CA, USA) as previously described [19]. DNA samples were bisulfite converted using the EZ DNA Methylation Gold Kit (Zymo Research Corporation; D5005) according to the manufacturer’s instructions. The targeted regions are indicated in the corresponding figures. Primers will be provided upon request.

### 2.6. Total RNA Isolation

Total RNA from frozen tissues and cultured cells was isolated using The Maxwell^®^ RSC simplyRNA Tissue Kit in the Maxwell RSC 48 Instrument (Promega; AS1340; Madison, WI, USA). RNA samples were treated with DNase to degrade all possible traces of contaminating genomic DNA (gDNA). RNA concentration was quantified using a NanoDrop spectrophotometer.

### 2.7. RT-PCR and qPCR

Reverse transcription followed by real-time polymerase chain reactions (PCRs) were performed using an iCycler (Bio-Rad, Hercules, CA, USA) and the iQ SYBR Green Supermix (Bio-Rad; #1708880). To monitor the specificity, the final PCR products were analyzed by melting curves and the amount of each transcript was expressed relative to the housekeeping gene ribosomal protein large P0 (*RPLP0*) as 2ΔCt, where ΔCt represents the difference in threshold cycle between the control and target genes. The sequence of primers used in the study will be provided upon request.

### 2.8. Prediction of lncRNA Function

To predict the function of the selected lncRNAs, we performed a guilt-by-association analysis (GBA) [20] using lncGSEA package version 0.1.0 in R version 3.6.3. Briefly, as described in the publication [20], lncGSEA prepares the input of expression matrices for lncRNA and coding genes in this case using the LIHC cohort from TCGA. Then, lncGSEA divides patients into two groups with high (top quartile) and low (bottom quartile) expression of the target lncRNA. Next, a differential expression analysis for coding genes is performed between the two groups of patients to determine the log2 fold change of each gene as the ranking metric. Finally, the ranked gene list is processed by fast gene set enrichment analysis (GSEA). The output is a matrix of the association of the target lncRNA with each gene set.

### 2.9. Statistical Analysis

Statistically significant differences were estimated using the GraphPad Prism software (version 8). A descriptive analysis was carried out to analyze the distribution of the samples with a D’Agostino normality test. A two-sided unpaired Student’s t-test or Mann–Whitney U-test were used according to sample distribution. All experiments were performed at least three times in duplicate. Correlation analysis between the expression of the Top35 LNDH and the Hsiao signature was performed by the non-parametric Spearman’s rank correlation test. Fisher’s test or the chi-squared test were used for comparison of demographic and clinical parameters. Kaplan Meier Curves were generated using GraphPad Prism software and Gene Expression Profiling Interactive Analysis (GEPIA) (webpage: http://gepia.cancer-pku.cn/ accessed on 1 December 2021), and a log-rank test was calculated. Statistical significance is indicated by *, *p* < 0.05; **, *p* < 0.01; ***, *p*< 0.001; ****, *p* ≤ 0.0001. ns indicates nonsignificant differences.

## 3. Results

### 3.1. Identification of a Set of lncRNAs Downregulated in HCC

**Figure 1:**
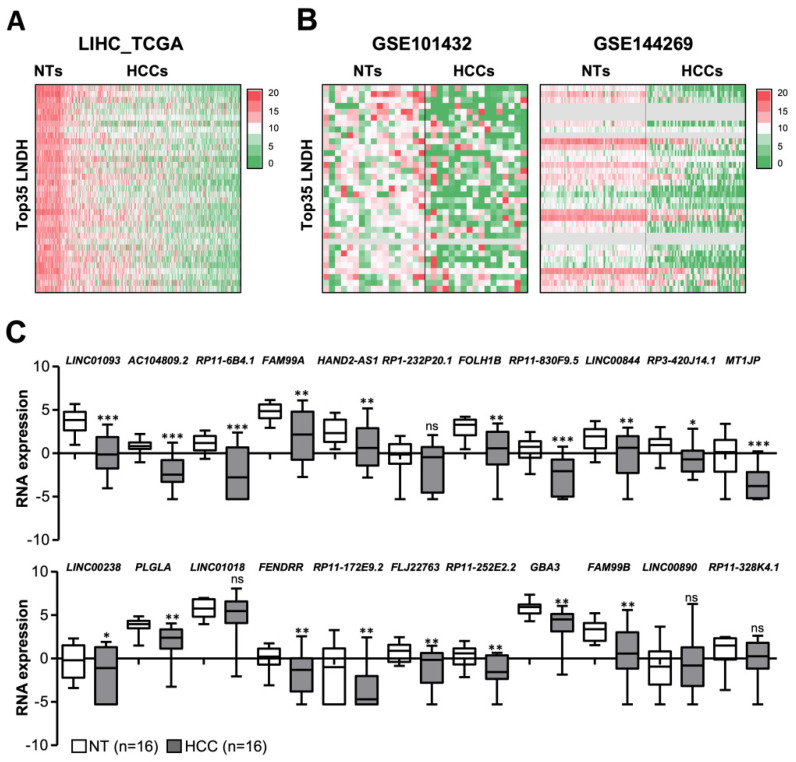
Top-ranked 35 lncRNAs downregulated in HCC (Top35 LNDH). (**A**,**B**) Heatmap reporting the expression levels of each of the Top 35 lncRNA (Top35 LNDH) in peritumoral and HCC tissue samples based on RNA-seq data from (**A**) LIHC TCGA (50 NTs and 374 HCCs), (**B**) GSE101432 (17 NTs and 17 HCCs) and GSE144269 (70 NTs and 70 HCCs) cohorts. The 35 lncRNAs are ranked according to their log2FC in the LIHC TCGA cohort, being the first one the lncRNA most downregulated. The color scale bar is shown on the right and represents relative expression. Grey color: data not available. (**C**) Transcript levels (from RNA-seq data expressed as log CPM) of the indicated 22 lncRNAs evaluated in paired peritumoral and tumor samples from a cohort of 16 HCC patients. The result of the statistical analysis is indicated for each lncRNA (HCC versus NT). The U the Mann-Whitney tests were used for statistical analysis. * *p* < 0.05, ** *p* < 0.01, *** *p* < 0.001, ns: nonsignificant differences.

Using RNA-seq data from TCGA, we previously identified deregulated lncRNAs in different tumour types, including HCC [11]. In contrast to other studies, the lists of differentially expressed genes for each tumor type were defined as those transcripts with an FDR < 1% after performing 200 comparisons between all peritumoral samples and the same number of tumor samples chosen randomly. For instance, in the case of HCC (LIHC), 200 comparisons of the 50 peritumoral liver samples with 50 HCC tissues randomly chosen from the 374 available were performed. This approach allows the retention of those genes with a higher probability of being deregulated in each tumour type [11]. From these analyses, we focused on the top-ranked 35 lncRNAs downregulated in HCC (Top35 LNDH), which met the criteria of logFC < −3.5 and FDR < 0.05 (Figure 1A and Table S1). The downregulation of most of these lncRNAs was also observed in other HCC cohorts with available RNA-seq data (GSE101432 and GSE144269 containing 17 and 70 paired HCC and adjacent liver samples, respectively; Figure 1B).

Accordingly, we found that 20 out of the Top35 LNDH (highlighted in bold in Table S1) were among the 525 lncRNAs identified by Yang et al. downregulated in HCC by overlapping the predictions of three statistical methods (GFOLD, DESeq2 and Wilcoxon; n = 20 paired HCC peritumoral/tumoral samples, GSE77509; [21]). We then validated the in silico findings by RNA-seq analysis of 16 paired HCCs-peritumoral tissue samples. We were able to annotate 22 out of the Top35 LNDH and we confirmed that all but four lncRNAs (*RP1-232P20.1*, *LINC01018*, *LINC00890* and *RP11-328K4.1*) are significantly downregulated in HCC compared to non-tumoral tissue (Figure 1C).

### 3.2. Downregulation of the Top35 LNDH through Promoter DNA Methylation

**Figure 2:**
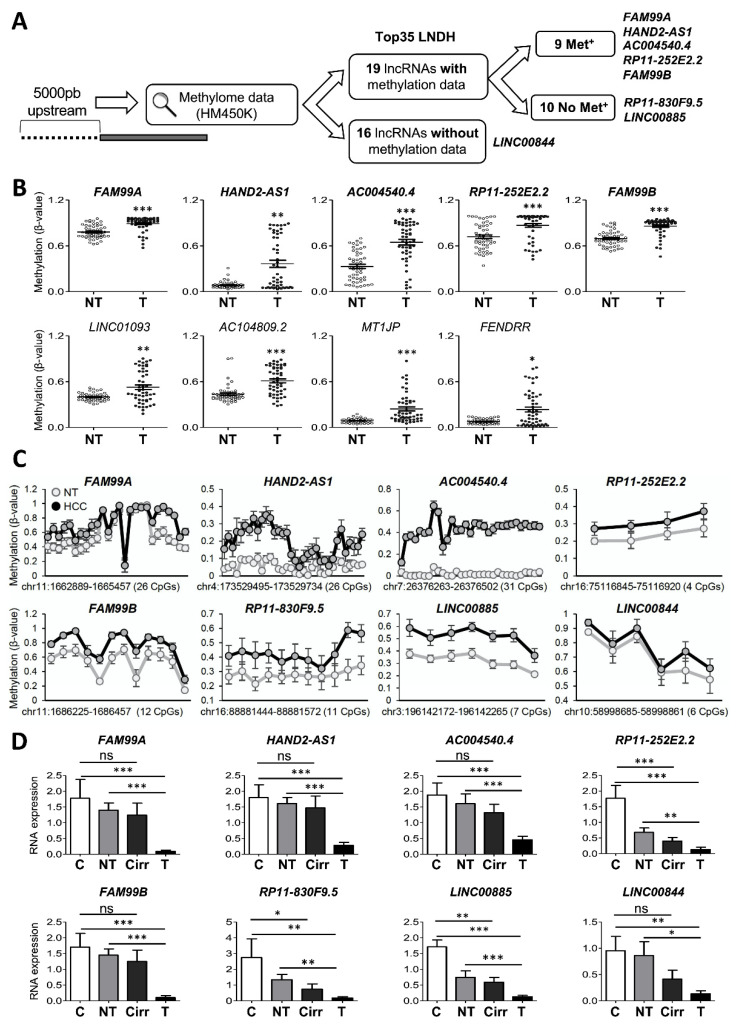
Evaluation of promoter DNA methylation levels of lncRNAs within the Top35 LNDH. (**A**) Schematic representation of the available methylation data for the promoter region (5000bp upstream lncRNA TSS) of the Top35 LNDH included in the Infinium HumanMethylation450 BeadChip (HM450K). Among the 19 lncRNAs with at least one promoter CpG analyzed in the array, 9 were hypermethylated (Met^+^). The eight lncRNAs selected for further validation by RT-qPCR and targeted bisulfite sequencing are indicated. (**B**) DNA methylation levels (β-values) of the most significantly hypermethylated CpG found in the promoter region of the mentioned nine lncRNAs in the 50 patients from the LIHC TCGA cohort for which both peritumoral (NT) and HCC tumor (T) samples were available. The average is indicated as a line. (**C**) Graphs reporting DNA methylation levels (β-values) obtained by targeted bisulfite sequencing of the promoter regions of the eight selected lncRNAs (see A) in peritumoral and HCC tissues (n = 7). Note that for all lncRNAs analyzed, HCC tissues were hypermethylated compared to paired non-tumoral tissues. (**D**) Expression level of the eight selected lncRNAs by RT-qPCR in healthy livers (C; n = 6), paired peritumoral and tumor samples (NTs and T; n = 10) and cirrhotic patients (Cirr; n = 12). The levels of *RPLP0* mRNA were evaluated and used as a reference to calculate the relative expression. The U the Mann-Whitney test was used for statistical analysis.* *p* < 0.05, ** *p* < 0.01, *** *p* < 0.001, ns: nonsignificant differences.

Since promoter DNA methylation is widely associated with gene silencing [22,23], including lncRNAs [24], we asked whether this epigenetic mechanism would be responsible for the downregulation of the Top35 LNDH in HCC. We first decided to analyze the methylation level of the CpGs located in the promoter region (5000bp upstream the lncRNA TSS) of these lncRNAs using the Infinium HumanMethylation450 BeadChip (HM450K) data from TCGA. Although this array covers over 450,000 methylation sites at single nucleotide resolution, only 2.9% of the total number of CpGs in the promoter regions of the Top35 LNDH were included. In fact, only 19 out of the 35 lncRNAs had at least one promoter CpG analyzed in the array, with a maximum of 10.4% of the promoter CpGs included (Figure 2A and Table S2). Although the promoter regions of these 19 lncRNAs were poorly represented, when compared the 50 HCC patients with available methylome data for both the peritumoral and the tumor tissue, we found at least one promoter CpG hypermethylated in the HCC tissue in 9 out of the 19 interrogated lncRNAs (Figure 2B). Similar results were observed when the methylome data of all the HCC tissues (n = 374) were compared to the 50 peritumoral samples (Figure S1). These findings suggest a negative correlation between DNA methylation and the expression level of this subset of the Top35LNDH.

We then decided to use targeted bisulfite sequencing to confirm the methylation of the Top35 LNDH, analyzing five lncRNAs found hypermethylated in the TCGA cohort (*FAM99A*, *HAND2-AS1*, *AC004540.4*, *RP11-252E2.2*, *FAM99B*), two lncRNAs with unmethylated covered CpGs (*RP11-830F9.5* and *LINC00885*) and one lncRNA not analyzed in the HM450K array (*LINC00844*) (Figure 2A). Importantly, bisulfite sequencing studies confirmed increased methylation levels of most CpGs analyzed within the promoter region of the five lncRNAs already identified to be hypermethylated in the TCGA cohort and similarly revealed higher methylation levels in the promoter CpGs of *RP11-830F9.5*, *LINC00855* and *LINC00844* in HCC samples compared to paired non-tumoral tissues (n = 7; Figure 2C). Accordingly, the expression levels of all eight lncRNAs were significantly downregulated in HCC samples compared to control, paired non-tumoral and cirrhotic liver tissues (Figure 2D). Moreover, *RP11-830F9.5*, *RP11-252E2.2* and *LINC00885* were already significantly downregulated in cirrhotic tissues compared with normal livers (Figure 2D).

**Figure 3:**
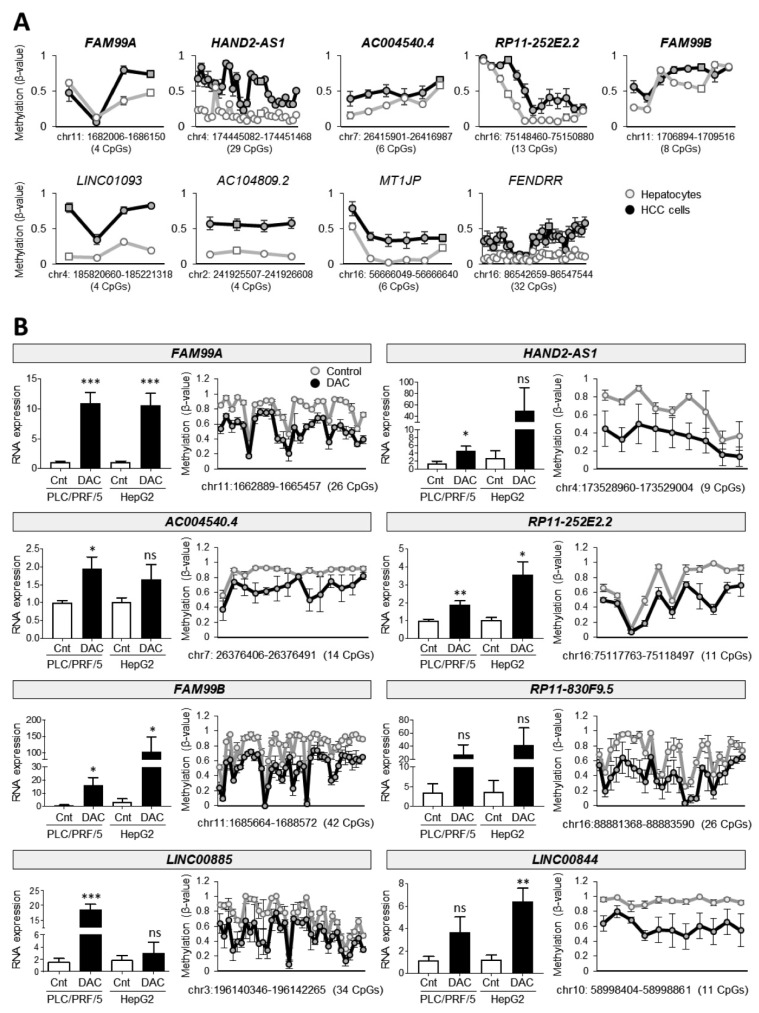
Evaluation of promoter DNA methylation levels of lncRNAs within the Top35 LNDH in human HCC cell lines. (**A**) DNA methylation levels (β-values) in HCC cell lines (n = 9; FLNEO, H801, HCO2, Hep3B, Huh75, LH86, SNU423, SNU449 and HepG2) and primary hepatocytes (n = 17) from GSE60753, of the CpGs analyzed by the HM450K array within the promoter region of the nine lncRNAs found hypermethylated in human HCC tissues (Figure 2B). The CpGs showed in Figure 2B are indicated as squares. (**B**) For the eight selected lncRNAs, graphs on the left report the expression levels by RT-qPCR in PLC/PRF/5 and HepG2 cells control or treated with 10 µM of 5-Aza-2′-deoxycytidine (DAC) for seven days. *RPLP0* expression was used as a housekeeping gene. At least three independent experiments in duplicate were performed for each cell line. Graphs on the right report DNA methylation levels (β-values) obtained by targeted bisulfite sequencing in PLC/PRF/5 cells control or treated with 10 µM of 5-Aza-2′-deoxycytidine (DAC) for seven days. The Mann-Whitney U test was used for statistical analysis. * *p* < 0.05, ** *p* < 0.01, *** *p* < 0.001, ns: nonsignificant differences.

To better characterize the regulation of these lncRNAs expression by DNA methylation, we used human HCC cultured cells. Firstly, analyzing available methylome data (GSE60753) from HCC cell lines (FLNEO, H801, HCO2, Hep3B, Huh75, LH86, SNU423, SNU449 and HepG2) and primary hepatocytes, we confirmed the hypermethylation of the available lncRNAs promoter CpGs found hypermethylated in TCGA HCC patients (Figure 3A) in human HCC cell lines compared to primary hepatocytes. We then verified the high methylation levels of the eight previously selected lncRNAs (Figure 2A) in the human HCC cell line PLC/PRF/5 (not included in the in silico analysis mentioned above by targeted bisulfite sequencing; Figure 3B). Importantly, treatment of both PLC/PRF/5 and HepG2 cell lines with the demethylating agent DAC drastically induced the expression of the eight lncRNAs (Figure 3B, left panels), in parallel to the decreased methylation level of their promoters (Figure 3B, right panels), demonstrating that DNA hypermethylation is responsible of the lncRNA downregulation observed in HCC cells.

### 3.3. The Top35 LNDH Are Preferentially Expressed in Adult Liver Tissue

**Figure 4:**
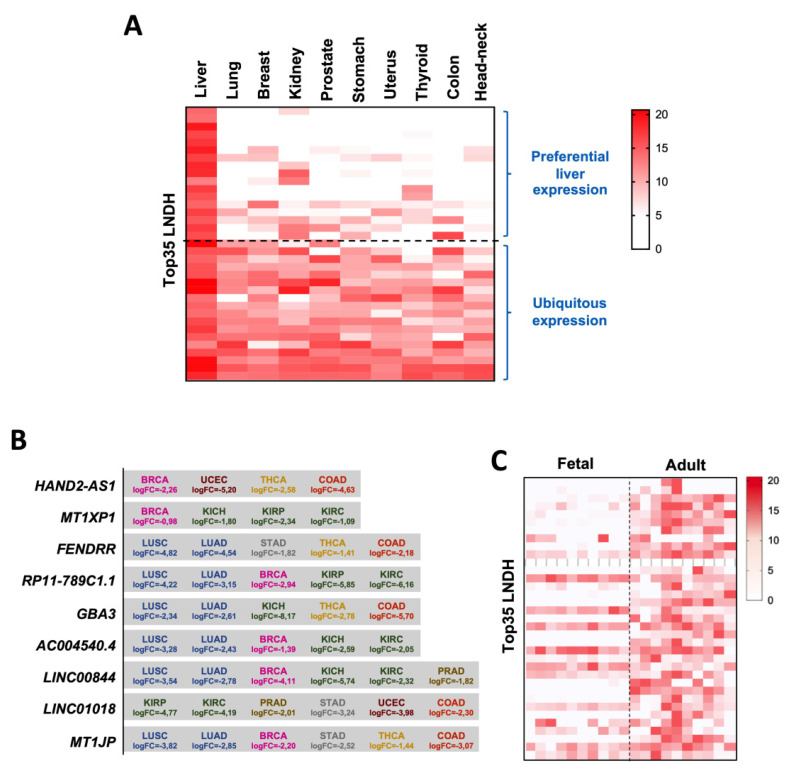
The Top35 LNDH can be divided into two subgroups according to their pattern of expression: preferentially expressed in adult liver and ubiquitously expressed. (**A**) Heatmap reporting the expression levels of each of the Top35 LNDH in control tissues of different origins from TCGA datasets. Tissue represented: liver (n = 50), lung (LUAD n = 59; LUSC n = 49), breast (n = 113), kidney (KICH n = 24, KIRC n = 72, KIRP n = 32), prostate (n = 52), stomach (n = 32), uterus (n = 35), thyroid (n = 58), colon (n = 41) and head-neck (n = 44). The 35 lncRNAs are ranked according to the number of types of tissues where they are expressed, 17 of them being preferentially expressed in liver, whereas the other 18 showed a ubiquitous expression. The color scale bar is shown on the right representing relative expression. The corresponding ranked list is shown in Supplementary Figure S2A. (**B**) List of the 9 lncRNAs significantly downregulated in more than four tumor types besides HCC. The tumor type in which each lncRNA is significantly downregulated and the corresponding log2FC are indicated. (**C**) Heatmap reporting the expression levels of each lncRNA from the Top35 LNDH in fetal and adult liver from GSE111845 (n = 10) ranked as in Figure 4A. The color scale bar is shown on the right representing relative expression.

It has been shown that most tumors downregulate lncRNAs preferentially expressed in their tissue of origin [11], therefore we asked whether theTop35 LNDH were preferentially expressed in liver tissue compared to other organs. For this, we compared the TCGA RNA-seq expression data in 12 different organs with at least 20 peritumoral samples available. The datasets analyzed were those from lung squamous cell carcinoma (LUSC), lung adenocarcinoma (LUAD), breast carcinoma (BRCA), chromophobe renal cell carcinoma (KICH), clear cell renal carcinoma (KIRC), papillary renal cell carcinoma (KIRP), prostate adenocarcinoma (PRAD), stomach adenocarcinoma (STAD), uterine corpus endometrial carcinoma (UCEC), thyroid carcinoma (THCA), colon adenocarcinoma (COAD) and head and neck squamous cell carcinoma (HNSC). For this analysis, the peritumoral samples of the two types of lung cancer (LUSC and LUAD) and the three types of kidney cancer (KICH, KIRC and KIRP) in the TCGA studies were pooled together. We confirmed that the Top35 LNDH were preferentially expressed in the liver compared to other organs, yet several of them were also expressed in other tissues (Figure 4A and Figure S2A). In fact, 17 lncRNAs showed a preferential hepatic expression, some of them being almost liver specific (Figure S2B), whereas the expression of the other 18 was more ubiquitous, being expressed in more than three tissues at similar levels than in the liver. Interestingly, regarding the 18 lncRNAs we found expressed in other tissues (Figure 4A), 15 were significantly downregulated in at least one other tumor type beside HCC, highlighting nine lncRNAs that were significantly downregulated in more than three tumor types beside HCC (Figure 4B).

Therefore, we analyzed the methylation status of the downregulated lncRNAs included in the HM450K array in the TCGA data from lung (LUSC and LUAD) and breast (BRCA) tumors, and we found that as in HCC, the promoter regions of those lncRNAs were hypermethylated in the tumour tissues compared to paired peritumoral samples (Figure S2C). Moreover, treatment of the lung cancer cell line H358 with the demethylating agent DAC significantly induced the expression of *AC004540.4* (Figure S2D). Altogether these findings suggest that DNA hypermethylation could be a general mechanism responsible for the downregulation of the Top35 LNDH in tumors, and this strengthens the relevance of epigenetic regulation of lncRNA gene expression and its alterations in carcinogenesis.

In view of the preferential expression of the Top35 LNDH in liver compared to other organs, we decided to analyze their expression during liver development. Using public RNA-seq data (n = 10; GSE111845), we found that the expression of most of the Top35 LNDH was markedly reduced in the fetal liver compared to the adult liver (Figure 4C), suggesting that their expression is important for the acquisition of the adult liver identity.

Altogether, these findings highlight a set of the Top35 LNDH preferentially expressed in the healthy adult liver downregulated through DNA methylation in HCC tissues. Moreover, some of them are also downregulated in other types of tumors, suggesting a role as tumor suppressor genes.

### 3.4. A Subset of the Top35 LNDH Play a Role in Liver Differentiation

**Figure 5:**
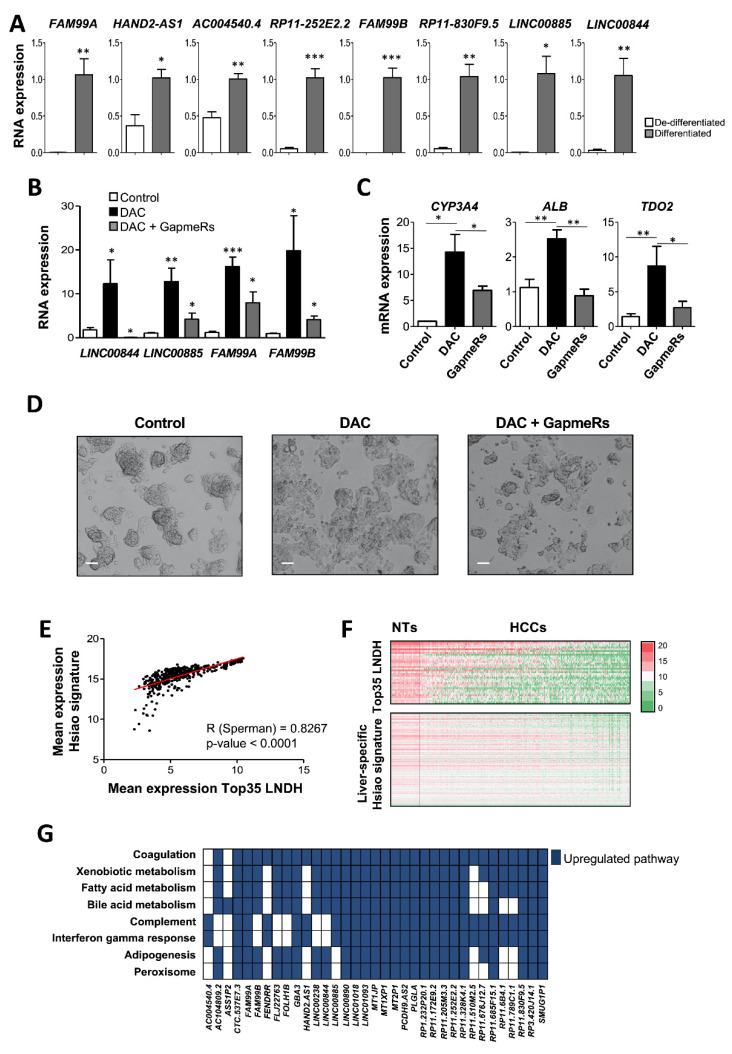
The Top35 LNDH participate in liver differentiation. (**A**) Expression levels by RT-qPCR of the 8 selected lncRNAs in de-differentiated human hepatoma cell line HepaRG compared to hepatocyte-like well-differentiated HepaRG cells. Experiments were performed at least three times in duplicate. (**B**,**C**) Histograms reporting *LINC00844*, *LINC00885*, *FAM99A*, *FAM99B* (**B**), *CYP3A4*, *ALB* and *TDO2.* (**C**) mRNA level by RT-qPCR in HepG2 cells after three days cultured with and without 10 µM *of* 5-Aza-2′-deoxycytidine (DAC), or after treatment with 10 µM *DAC* simultaneously to the transfection with a combination of specific GapmeRs for *LINC00844, LINC00885, FAM99A* and *FAM99B*. *RPLP0* expression was used as a housekeeping gene. All experiments were performed at least three times in duplicate. (**D**) Representative images of HepG2 cells three days after culture without (control) or with 10 µM of 5-Aza-2′-deoxycytidine (DAC), or after treatment with 10 µM DAC simultaneously to the transfection with a combination of specific GapmeRs for *LINC00844*, *LINC00885*, *FAM99A* and *FAM99B*. Scale bar 20µm. (**E**) Graph reporting the positive correlation between the mean expression of the Top35 LNDH and the mean expression of Hsiao liver specific signature (249 liver-specific genes) in the 374 HCC patients from the LIHC TCGA cohort. (**F**) Heatmap reporting the expression levels of each lncRNA from the Top35 LNDH and each gene from the Hsiao liver specific signature in the 50 peritumoral and 374 HCCs from the LIHC TCGA cohort. Patients are organized according to the highest (left) to the lowest (right) mean expression of the Top35 LNDH. The color scale bar is shown on the right (expressed as relative expression). (**G**) GBA analysis of the Top35 LNDH. For each lncRNA, the upregulated pathways (FDR<0.05) are shown in dark blue. The Mann-Whitney U test was used for statistical analysis. * *p* < 0.05, ** *p* < 0.01, *** *p* < 0.001.

At this point we reasoned that the expression of the Top35 LNDH could be associated with the grade of liver differentiation. To validate this hypothesis, we used a well-established protocol of in vitro hepatocyte-like differentiation of the human hepatoma cell line HepaRG [18]. Efficiency of hepatocyte differentiation was validated by evaluating the typical morphological changes (Figure S3A) and expression of hepatic-specific genes (Figure S3B). We therefore evaluated the expression levels of the eight selected Top35 LNDH in differentiated and de-differentiated HepaRG cells and found a significant increased expression of all lncRNAs after the induction of HepaRG differentiation (Figure 5A). These results demonstrated that the expression of these lncRNAs positively correlates with the grade of liver differentiation.

To decipher whether this was a mere association or if the expression of these lncRNAs was indeed necessary for the transcription of the hepato-specific genes, we used a second in vitro model of hepatic differentiation. It has been well described that the inhibition of DNA methylation is associated with a significant restoration of liver differentiation [25,26,27,28]. Using specific GapmeRs in HepG2 cells, we prevented the induction of *LINC00844*, *LINC00885*, *FAM99A* and *FAM99B* observed upon treatment with DAC (Figure 5B). Importantly, we found that when the expression of the four selected lncRNAs was inhibited by the combination of the specific GapmeRs, the expected induction of expression upon treatment with DAC of the promoter-hypermethylated liver specific genes *CYP3A4, ALB* and *TDO2* was significantly reduced (Figure 5C). Moreover, and accordingly, the morphological changes induced in HepG2 cells upon DAC treatment, were less evident when the selected 4 lncRNAs were silenced by the combination of GapmeRs (Figure 5D).

To further support the relationship of the Top35 LNDH expression with liver differentiation, we took advantage of a list of 249 “liver-specific genes” defined by Hsiao and colleagues [29] and observed a significant positive correlation between the level of expression of the Top35 LNDH and Hsiao liver specific signatures in the LIHC RNA-seq data from TCGA (Sperman R = 0.8267 and *p*-value = 0.0001; Figure 5E,F). Moreover, in an attempt to predict the function of this set of lncRNAs, we performed a GBA analysis [20]. The results indicated that the Top35 LNDH expressed in healthy adult liver and downregulated in HCC tissue positively correlated with the expression of genes associated with typical liver specific functions such as coagulation, xenobiotic metabolism, fatty acid metabolism, bile acid metabolism or adipogenesis (Figure 5G).

Altogether, these results suggest that the Top35 LNDH are related with liver differentiation, and accordingly we have demonstrated that at least a subset of four of those lncRNAs are required to maintain a liver differentiated transcriptome.

### 3.5. The Expression of the Top35 LNDH Correlates with Tumor Grading and Patients’ Overall Survival

We therefore decided to evaluate the association between the level of expression of the Top35 LNDH and the grade of tumor differentiation in human samples. We first analyzed the expression levels of the Top35 LNDH in the TCGA HCC patients divided in four groups according to their histological grade (G1 to G4) and found that their mean expression levels significantly decreased as the degree of de-differentiation increased (Figure S4A).

**Figure 6:**
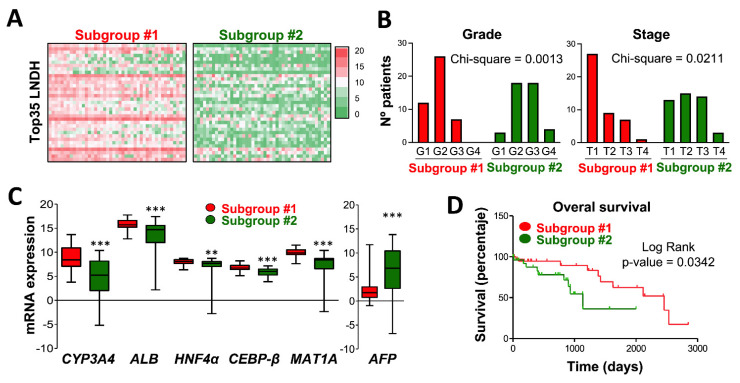
The Top35 LNDH correlates with tumor differentiation and patient survival. (**A**) Heatmap reporting the expression levels of each lncRNA from the Top35 LNDH in the two HCC subgroups established from the LIHC TCGA cohort: Subgroup #1 (45 patients) with higher level of expression of Top35 LNDH and Subgroup #2 (45 patients) with lower level of expression of Top35 LNDH. The color scale bar is shown on the right representing relative expression. (**B**) Number of patients at the different histological grade (G1 to G4) and pathological stage (T1 to T4) in each subgroup defined as in A. (**C**) Box plots reporting the mRNA expression levels of *CYP3A4*, *ALB*, *HNF4α*, *C/EBP-β*, *MAT1A* and *AFP* obtained from the LIHC TCGA cohort in the two HCC subgroups established as described in A (Subgroup #1 in red and Subgroup #2 in green). The U the Mann-Whitney test was used for statistical analysis. (**D**) Kaplan-Meier curves reporting the overall survival of the two HCC subgroups established as described in A. ** *p* < 0.01, *** *p* < 0.001.

To decipher the clinical relevance of Top35 LNDH expression, we divided HCC TCGA patients in two subgroups according to the level of expression of the Top35 LNDH. Subgroup #1 was formed by the 45 patients with the highest level of expression and Subgroup #2 was formed by the 45 patients with the lowest level of expression (Figure 6A). Interestingly, we found that in agreement with the methylation dependent downregulation of the Top35 LNDH, patients in Subgroup #2 showed significantly higher methylation levels in 7 out of the nine lncRNAs analyzed in Figure 2B and increased expression levels of the three DNA methyltransferases (*DNMT1*, *DNMT3A* and *DNMT3B*) compared with Subgroup #1 (Figure S4B,C). We then compared the demographic and clinical parameters of patients in Subgroup #1 and Subgroup #2. We did not observe any significant association with risk factors; however, our data demonstrated a significant enrichment of Caucasian males in Subgroup #1 and Asian females in Subgroup #2 (Figure S4D). More intriguing, Subgroup #2 with a lower expression of Top35 LNDH was significantly enriched in patients with a higher histological grade (G3 (poorly differentiated) and G4 (undifferentiated)) and pathological stage (T3 and T4) compared with Subgroup #1 (Figure 6B). Accordingly, Subgroup #2 had lower levels of expression of liver specific genes such as *CYP3A4*, *ALB*, hepatocyte nuclear factor 4 alpha (*HNF4α*), CCAAT/enhancer-binding protein beta (*C/EBP-β*) and methionine adenosyltransferase 1A (*MAT1A*), together with higher levels of expression of alpha-fetoprotein (*AFP*) (Figure 6C). Finally, and relevantly, our results showed that lower levels of expression of the Top35 LNDH (Subgroup #2) were significantly associated with a poorer overall survival (log rank *p*-value = 0.0342; Figure 6D). Interestingly, when we individually analyzed the level of expression of each Top35 LNDH we found that 16 out of the 35 lncRNAs were significantly correlated with overall survival rate (Figure S5), suggesting that their expression could be a good prognostic marker.

All in all, our results identify a relevant set of lncRNAs (Top35 LNDH) implicated in the regulation of liver specific genes expression and hepatic differentiation, which are downregulated during hepatocarcinogenesis by DNA methylation in relation with patients’ prognosis.

## 4. Discussion

Epigenetic mechanisms, including DNA methylation, post-translational histone modifications, non-coding RNAs (ncRNAs) and 3D genome structure [6] tightly control gene expression in a cell-type and dynamic manner. Epigenetic modifications are thus critical in organism development, dictating cell lineage choices and later maintaining the transcriptomic landscape towards a terminally differentiated state [30]. Because of this epigenetic fine-tuned control of gene expression, alterations of the epigenetic mechanisms are central events in tumor initiation that are known to impact on all hallmarks of cancer [8,31]. For instance, it has been described that dysregulated DNA methylation is one of the early events of carcinogenesis, reprograming gene expression profiles and increasing chromosomal instability [32,33]. Moreover, in the last years, the dysregulation of the levels of several lncRNAs has also been involved in the development and progression of cancer [34,35]. In this regard, in a previous work, using pan-cancer comparison we identified a list of lncRNAs deregulated in several tumor types [11]. We found that, compared to mRNA, lncRNAs were deregulated in a more tumor-specific manner and that upregulated lncRNAs in tumors were preferentially expressed in the testis, brain, digestive tract or blood/spleen healthy tissues [11]. In the present work, we were interested in characterizing the top-ranked 35 lncRNAs downregulated in HCC (Top35 LNDH). First, we have validated their downregulation in two additional in silico and another experimental HCC cohort and confirmed their higher expression not only in the peritumoral tissue, but also in the healthy liver. In fact, some of the lncRNAs tested were already downregulated in the peritumoral and cirrhotic tissue compared with the normal livers, in line with the pre-neoplastic condition of cirrhosis and highlighting an early role of those lncRNAs in liver carcinogenesis [36]. The validity of our system of differential gene expression selection and the relevance of the selected Top35 LNDH is supported by the fact that some of them have been identified as deregulated in HCC by others, in high-throughput [21,37,38] or individual studies (*LINC01093* [39,40], *FAM99A* [41], *FAM99B* [42], *HAND2-AS1* [43,44], *LINC00844* [45,46] or *GBA3* [47]) being described as cell proliferation inhibitors, prognostic indicators, or potential biomarkers in HCC. Moreover, a set of Top35 LNDH is also downregulated in other tumor types, mainly lung and breast tumors, suggesting a more pan-cancer role as tumor suppressor genes. In this regard, and in agreement with our findings, *HAND2-AS1* [48], *FENDERR* [49,50], or *MT1JP* [51,52], among others, have already been reported as downregulated in different types of tumors.

We hypothesized that the Top35 LNDH may be downregulated by a common mechanism. In this regard, the DNA methylation landscape of cancer cells is generally characterized by an abnormal expression of the DNA methyltransferases, a diffuse DNA hypomethylation and a focal hypermethylation of CpG islands present in about 70% of human gene promoters [23,53]. This promoter hypermethylation is associated with an inhibition of transcription initiation [22,23]. Here we demonstrate, using in silico methylome data, targeted bisulfite sequencing and in vitro experiments in cultured cells treated with the demethylating agent DAC, that the decreased expression of these Top35 LNDH in HCC is due to promoter DNA hypermethylation. In support of these findings, we observed that HCC patients with lower levels of the Top35 LNDH have higher methylation levels in the promoter of these lncRNAs and increased levels of *DNMT1*, *DNMT3A* and *DNMT3B* expression than patients with higher levels of Top35 LNDH. Moreover, and accordingly, a significant correlation between DNA methylation and the level of expression of six (*LINC01093*, *AC104809.2*, *FAM99A*, *HAND2-AS1*, *AC004540.4* and *FLJ22763*) out of the Top35 LNDH in HCC has been recently reported [21,54]. In essence, and as mentioned above, both DNA methylation and lncRNAs represent epigenetic mechanisms required to regulate gene expression and specific phenotypes [30]. Therefore, the coordinated silencing of the Top35 LNDH could have a role in regulating gene expression to favor hepatocarcinogenesis.

The identity of a fully differentiated cell is based on a specific transcriptome which is progressively acquired throughout fetal development and must be maintained throughout adulthood to ensure the correct function of the cell [1]. In fact, the capability to evade or escape from the state of terminal differentiation is now recognized as a critical component of cancer pathogenesis [55]. Hepatocellular identity and function include the expression of transcription factors such as HNF 1 and 4 alpha (*HNF1α* and *HNF4α*) and *C/EBP α* and *β*, as well as metabolic proteins and enzymes such as albumin, cytochrome P450 (CYP) isoforms, fructose-1,6-bisphosphatase 1 (*FBP1*) or *MAT1A*, among others [1]. In this study we have found that the expression of the Top35 LNDH is markedly induced during liver development, similarly to other hepato-specific genes [1]. Moreover, and in agreement with the notion that compared to mRNAs, lncRNAs are more tissue specific [11,56], we observed that a set of the Top35 LNDH was preferentially expressed in the liver compared to other tissues. These findings suggested that the expression of the Top35 LNDH could be important for adult liver identity. The notion that the Top35 LNDH positively correlates with the grade of liver differentiation was further demonstrated by: (i) their higher expression in the HepaRG cell line after induction of hepatocyte-like differentiation; (ii) the positive correlation observed in the liver of patients between the level of expression of the Top35 LNDH and genes associated with typical liver specific functions or the expression of the liver specific gene signature described by Hsiao et al. [29]; and (iii) by the negative correlation between the expression of the Top35 LNDH and tumor grading in HCC patients.

In fact, our results demonstrate that the expression of the Top35 LNDH not only parallels hepatic differentiation, but is required for the expression of hepato-specific genes. We found that the knockdown of a pool of 4 Top35 LNDH impaired the induction of *CYP3A4* and *ALB* expression elicited by a DNA demethylation agent. Indeed, the differentiated phenotype is governed by a specific epigenetic landscape that determines the expression of the distinct cell-type set of genes [5,9], and the mechanism of action of some epigenetic drugs is associated with the restoration of liver differentiation [25,26,27]. A hypermethylation of *CYP3A4*, *ALB* and *FBP1* promoter regions, among others, occurs in HCC cells, and demethylating treatments result in significant promoter demethylation accompanied by the induction of their expression levels [25,26]. Here, we demonstrate that the expression of the Top35 LNDH is also regulated by DNA methylation, and their induction is in turn required for the demethylation dependent induction of at least *CYP3A4* and *ALB*. Further studies are required to finely decipher the molecular mechanism implicated in this dependency. Although few examples are yet available in this context, an elegant report highlighting the potential complexity of these mechanisms has described that in hepatoma cells the lncRNA SNHG6 is a negative regulator of MAT1A protein expression by triggering the miR1297/FUS pathway to regulate nucleocytoplasmic shuttling of *MAT1A* mRNA, while activating *MAT2A* mRNA expression by suppressing the direct binding of miR-1297 to *MAT2A* 3′UTR mRNA [57]. In any case, and altogether, our results demonstrate that a coordinated chain of epigenetic events, involving DNA demethylation and the expression of lncRNAs, is required to maintain the transcriptome responsible for liver identity.

From a clinical point of view, the loss of liver identity and the degree of differentiation of hepatic cells have an impact on patient management and prognosis [2,3,4]. Accordingly, we found that HCC patients with the lowest level of expression of the Top35 LNDH had a significantly worse prognosis than patients with higher level of Top35 LNDH. Interestingly, the individual expression of a large number of these lncRNAs correlate with overall survival, suggesting that the use of their expression to predict patients’ progression deserves further study. Moreover, the successful management of cancer patients depends on their early diagnosis and effective therapy. As mentioned above, the mechanism of action of some epigenetic drugs is based on their differentiation effect [25,26,27,28]; therefore, more-differentiated tumors will show limited response. Further evidence is required to confirm whether, as suggested, the level of expression or the methylation status of any or all the Top35 lncRNAs identified could be used as biomarkers for targeted therapy.

## 5. Conclusions

The progression and prognosis of HCC patients negatively correlates with the grade of tumor dedifferentiation [2,3,4]. Multiple mechanisms govern the expression of genes which determine the fully differentiated cell state and, thus, its function [5]. Here, we identify a set of lncRNAs (Top35 LNDH) as part of the hepatic differentiated signature who’s expression is downregulated during the process of hepatocarcinogenesis by DNA methylation. Importantly the loss of this Top35 LNDH compromises the expression of other hepato-specific genes, and it is significantly associated with patient prognosis.

These data represent a new example of the complexity and interconnection between different epigenetic mechanisms involved in the regulation of gene expression and hepatic differentiation and the impact that their regulation can have in the progression of liver disease and the prognosis of HCC patients.

## Data Availability

Reagents and detailed methods of all procedures are provided in “Material and Methods” of this manuscript or cited accordingly. Other data, analytic methods, and study materials will be made available upon reasonable request from the corresponding authors.

## Supplementary Materials

The following supporting information can be downloaded at: https://www.mdpi.com/article/10.3390/cancers14092048/s1: Table S1: Information of the 35 top-ranked downregulated lncRNAs in HCC. Table S2: CpGs in the promoter regions of the Top35 LNDH included in HM450K. Figure S1: Promoter DNA methylation of a set of lncRNAs in human HCC tissues from TCGA database. Figure S2: The Top35 LNDH are preferentially expressed in adult liver tissue, yet a set of them expressed in other tissues are downregulated in the corresponding tumor type. Figure S3: Validation of the efficiency of hepatocyte-like differentiation of HepaRG cells. Figure S4: Correlations between the level of expression of the Top35 LNDH and the grade of tumor differentiation, DNA methylation and other patient characteristics from the LIHC TCGA cohort. Figure S5: Overall survival of the HCC patients from the LIHC TCGA cohort divided in two groups according to the level of each indicated lncRNA.
