# Supplementary material for: DNA Methylation Regulates a Set of Long Non-Coding RNAs Compromising Hepatic Identity during Hepatocarcinogenesis
Source: Cancers (Basel). 2022 Apr 19;14(9):2048. doi: 10.3390/cancers14092048 (PMC9102946; doi:10.3390/cancers14092048)
Supplement: Supplementary file 1 [file cancers-14-02048-s001.zip › cancers-1679988-supplementary.pdf]

DNA methylation regulates a set of long non-coding RNAs compromising hepatic identity during hepatocarcinogenesis

Miriam Recalde [1], María Gárate-Rascón [1], José María Herranz [1,2], María Elizalde [1], María Azkona [1],
Juan P. Unfried [3], Loreto Boix [2,4], María Reig [2,4], Bruno Sangro [2,5,6], Maite G. Fernández-Barrena [1,2,6], Puri Fortes [2,3,6], Matías A. Ávila [1,2,6], Carmen Berasain [1,2,6,*] and María Arechederra [1,2,6,*]

**Table S1: Information of the 35 top-ranked downregulated lncRNAs in HCC**							
gene_id	chr	start	end	strand	gene_name	FDR	logFC
ENSG00000267444.1	chr18	49650350	49651160	+	*SMUG1P1*	4,07048E-17	-5,843440201
ENSG00000249173.4	chr4	184893871	184899454	-	***LINC01093***	4,35079E-18	-5,81150037
ENSG00000223991.1	chr2	240981515	240986072	-	***AC104809.2***	8,5556E-10	-5,593080675
ENSG00000260015.1	chr16	71539834	71541825	-	*RP11-510M2.5*	4,4331E-16	-5,191284795
ENSG00000250266.1	chr4	170273919	170283079	+	***RP11-789C1.1***	4,22455E-13	-5,058994831
ENSG00000251049.2	chr4	57595940	57605872	-	***RP11-685F15.1***	1,92388E-22	-5,045660505
ENSG00000243694.2	chr3	80993868	81095647	+	*RP11-6B4.1*	3,36068E-11	-4,964447068
ENSG00000223922.1	chr2	38810432	38811654	-	*ASS1P2*	7,41816E-15	-4,825092135
ENSG00000228842.3	chr13	66825169	66915031	+	*PCDH9-AS2*	4,9245E-14	-4,571881343
ENSG00000205866.3	chr11	1665597	1667856	+	***FAM99A***	3,50822E-09	-4,536444248
ENSG00000237125.7	chr4	173527270	173591324	+	***HAND2-AS1***	1,17274E-14	-4,533179682
ENSG00000162840.4	chr4	68376323	68376505	+	*MT2P1*	4,76523E-13	-4,454857069
ENSG00000269985.1	chr6	5451683	5458075	-	***RP1-232P20.1***	1,33289E-10	-4,40102261
ENSG00000134612.10	chr11	89639227	89698718	+	*FOLH1B*	4,98141E-11	-4,332328133
ENSG00000259881.1	chr16	88881038	88887136	+	***RP11-830F9.5***	3,20445E-12	-4,258499339
ENSG00000237949.1	chr10	58999626	59001543	+	***LINC00844***	9,06972E-08	-4,213034295
ENSG00000215124.2	chr6	11861626	11862970	-	*RP3-420J14.1*	1,43893E-11	-4,120989162
ENSG00000255986.5	chr16	56635739	56637086	+	*MT1JP*	2,06541E-09	-4,08644754
ENSG00000233929.1	chr1	16241213	16241398	-	*MT1XP1*	4,11443E-12	-4,063211373
ENSG00000225792.1	chr7	26372144	26376701	-	***AC004540.4***	7,3372E-12	-4,056356393
ENSG00000196553.12	chr14	66486354	66498553	+	***LINC00238***	1,09727E-10	-4,000134521
ENSG00000240935.5	chr2	106382171	106393031	+	*PLGLA*	2,15484E-13	-3,986095023
ENSG00000250056.4	chr5	6582136	6588499	+	**LINC01018**	7,90117E-06	-3,980021903
ENSG00000224652.1	chr3	196142636	196160890	+	***LINC00885***	9,84296E-09	-3,945882399
ENSG00000268388.4	chr16	86474529	86509099	-	***FENDRR***	6,25893E-17	-3,935351931
ENSG00000248884.1	chr5	68430427	68434481	-	***CTC-537E7.3***	2,77532E-13	-3,897176724
ENSG00000230058.1	chr13	40343957	40350303	-	*RP11-172E9.2*	5,26034E-09	-3,895908186
ENSG00000262003.1	chr17	909632	911212	+	*RP11-676J12.7*	1,64009E-08	-3,894250224
ENSG00000241224.5	chr3	109118252	109150514	+	***FLJ22763***	6,06105E-09	-3,836028671
ENSG00000248319.1	chr4	169917761	169975902	-	***RP11-205M3.3***	4,02825E-10	-3,812141302
ENSG00000261058.1	chr16	75119558	75144200	+	***RP11-252E2.2***	5,08334E-12	-3,810663379
ENSG00000249948.5	chr4	22692914	22819575	+	*GBA3*	3,37976E-09	-3,763979338
ENSG00000205865.4	chr11	1683269	1685629	-	***FAM99B***	7,66579E-09	-3,757721587
ENSG00000260802.1	chrX	111511662	111522399	+	*LINC00890*	0,005505908	-3,713907204
ENSG00000248740.4	chr4	103256159	103453658	+	***RP11-328K4.1***	6,09337E-10	-3,712167612

Table S2: CpGs in the promoter regions of the Top35 LNDH included in HM450K

gene_name	IlmnID	chr	MAPINFO (hg19)		gene_name	IlmnID	chr	MAPINFO (hg19)
LINC01093	cg00705600	4	185820660		*FENDRR*	cg00606102	16	86542659
	cg20559943	4	185820756			cg26918279	16	86542693
	cg24975266	4	185821270			cg01168283	16	86542789
	cg06781910	4	185821318			cg06602857	16	86542807
AC104809.2	cg19721478	2	241925507			cg01145317	16	86542838
	cg22704351	2	241925548			cg05555338	16	86542864
	cg20971623	2	241925550			cg03366439	16	86542878
	cg24574692	2	241926608			cg01695533	16	86542905
RP11-6B4.1 (LINC02027)	cg25472614	3	81042167			cg20368988	16	86542943
FAM99A	cg14089103	11	1682006			cg06999379	16	86543092
	cg09512973	11	1682139			cg07769121	16	86543519
	cg04087449	11	1686117			cg06675147	16	86543532
	cg24218935	11	1686150			cg16630791	16	86543538
HAND2-AS1	cg08548444	4	174445082			cg09586080	16	86543543
	cg26463328	4	174445349			cg01142386	16	86543567
	cg11444009	4	174445870			cg03492747	16	86543808
	cg10852861	4	174446201			cg08157228	16	86544308
	cg04699272	4	174447055			cg25971347	16	86544339
	cg16538531	4	174447292			cg00314966	16	86544346
	cg01566965	4	174447847			cg27122536	16	86544658
	cg01967399	4	174448084			cg10514097	16	86544787
	cg27218796	4	174448128			cg08142918	16	86546374
	cg10933003	4	174448341			cg03697918	16	86546628
	cg24974365	4	174448549			cg24908603	16	86546631
	cg08297751	4	174448705			cg07056644	16	86546785
	cg05155840	4	174448841			cg27453745	16	86546938
	cg04771946	4	174449488			cg09338251	16	86546979
	cg16162058	4	174449827			cg06834912	16	86547203
	cg01580681	4	174450016			cg04787888	16	86547322
	cg15707093	4	174450353			cg01243371	16	86547386
	cg19178853	4	174450408			cg00551679	16	86547530
	cg10541864	4	174450722			cg02783918	16	86547544
	cg25102370	4	174451141		*FLJ22763*	cg14877963	3	108854657
	cg02774439	4	174451251			cg06183295	3	108855390
	cg21530280	4	174451394			cg04882739	3	108855428
	cg00443971	4	174451398		*RP11-205M3.3*	cg22259097	4	170897176
	cg03676512	4	174451413			cg07578108	4	170897214
	cg26650303	4	174451429			cg23879743	4	170897284
	cg13935962	4	174451443			cg03116035	4	170897498
	cg07410872	4	174451455		*RP11-252E2.2*	cg00004883	16	75148460
	cg05536800	4	174451460			cg06569826	16	75148635
	cg03905758	4	174451468			cg07565228	16	75148743
MT2P1	cg25976440	4	69239481			cg07475178	16	75149110
RP11-830F9.5 (AC092384.2)	cg02849719	16	88945618			cg16724332	16	75149501
	cg09928027	16	88945708			cg03991512	16	75150456
	cg02393091	16	88945894			cg07320140	16	75150611
	cg05370094	16	88946905			cg24429836	16	75150744
	cg08688816	16	88947068			cg16540789	16	75150784
	cg02192204	16	88947943			cg09899215	16	75150799
	cg02216032	16	88949367			cg03085549	16	75150819
	cg00402956	16	88949636			cg03743982	16	75150833
MT1JP	cg10638827	16	56665855			cg04906242	16	75150880
	cg05925949	16	56665870		*GBA3*	cg11210703	4	22693739
	cg02132560	16	56666049			cg20317116	4	22694230
	cg05581701	16	56666323		*FAM99B*	cg01291856	11	1706894
	cg00916884	16	56666334			cg26386189	11	1706902
	cg02160530	16	56666428			cg10557009	11	1707044
	cg07229186	16	56666575			cg08380262	11	1707163
	cg04994964	16	56666640			cg15832905	11	1707625
	cg04523867	16	56667262			cg05463727	11	1707647
	cg00170620	16	56669038			cg10702283	11	1709459
MT1XP1	cg22816278	1	16571023			cg11872086	11	1709516
AC004540.4	cg17035412	7	26415901		*RP11-328K4.1*	cg04100169	4	104177163
	cg14592933	7	26416122					
	cg26526379	7	26416184					
	cg17880050	7	26416735					
	cg12492504	7	26416890					
	cg24755163	7	26416987					
LINC01018	cg24542200	5	6580363					
	cg21768260	5	6581683					
	cg24189734	5	6581774					
	cg23517115	5	6581849					
	cg06872822	5	6582121					
	cg03550208	5	6582193					
LINC00885	cg13403369	3	195868640					

Supplementary figures

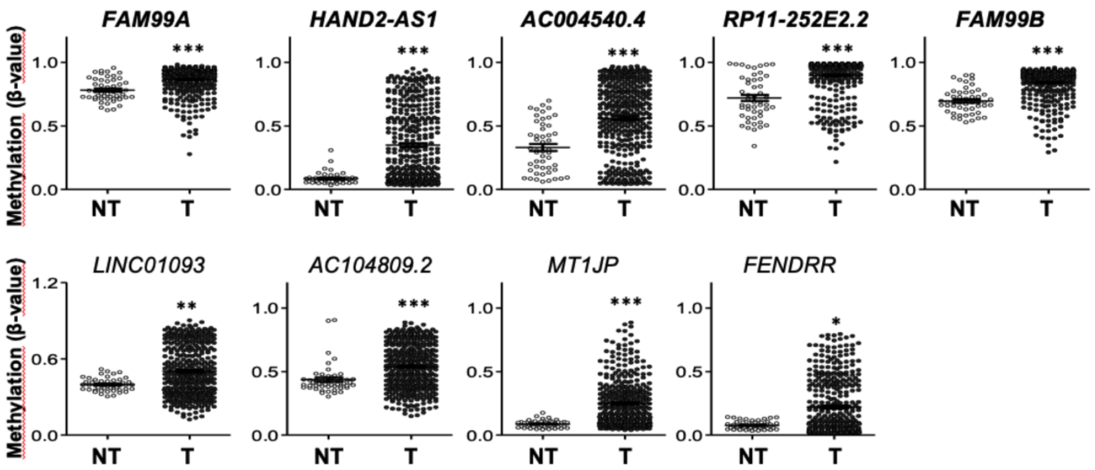

Figure S1. DNA methylation levels (β-values) of the CpG found most significantly hypermethylated in the promoter region of the indicated 9 lncRNAs in the 374 HCC tissues (T) and 50 peritumoral samples (NT). The average is indicated as a line. The U the Mann-Whitney test was used for statistical analysis.* $p < 0.05$, ** $p < 0.01$, *** $p < 0.001$.

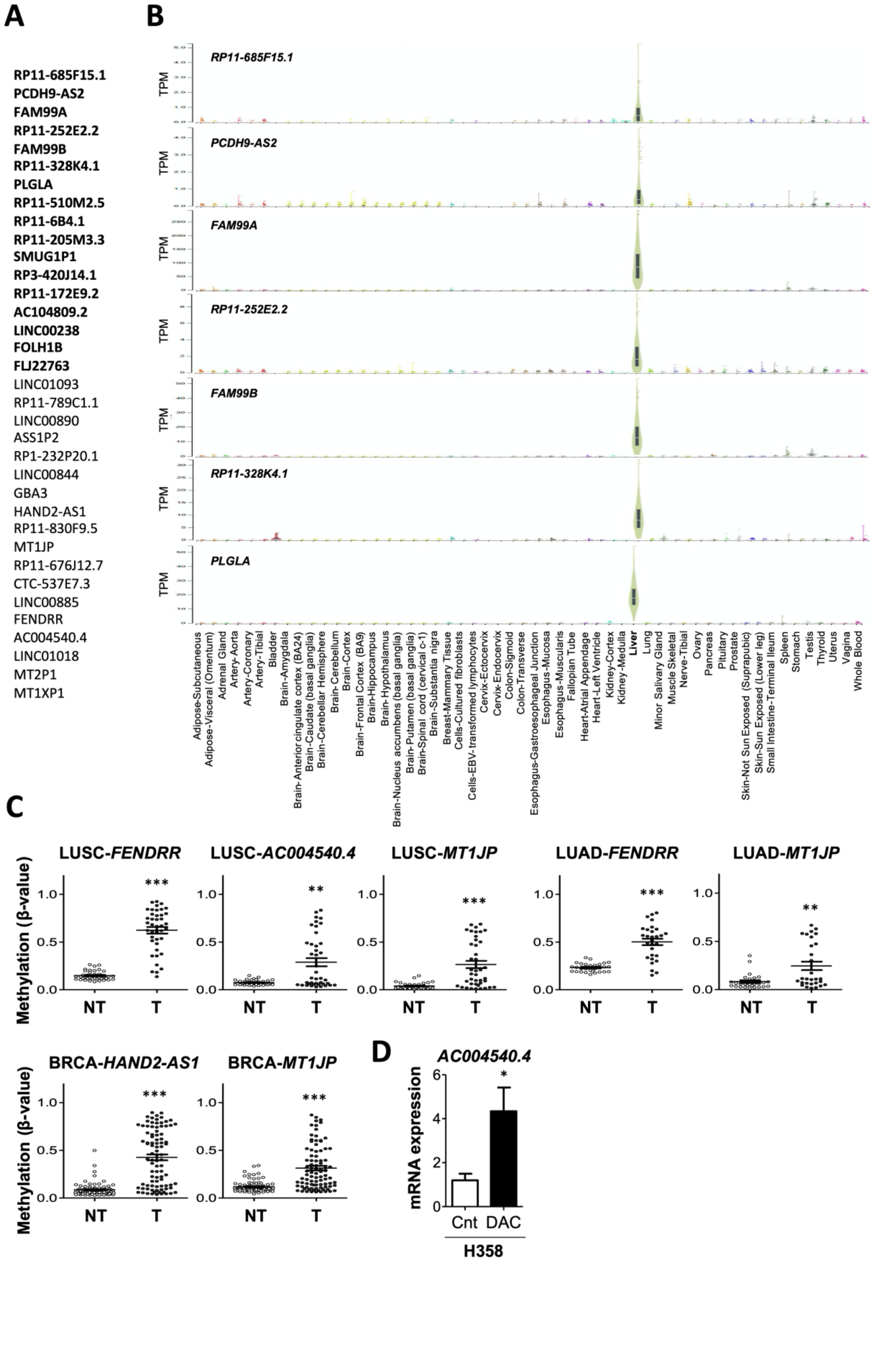
A
RP11-685F15.1
PCDH9-AS2
FAM99A
RP11-252E2.2
FAM99B
RP11-328K4.1
PLGLA
RP11-510M2.5
RP11-6B4.1
RP11-205M3.3
SMUG1P1
RP3-420J14.1
RP11-172E9.2
AC104809.2
LINC00238
FOLH1B
FLJ22763
LINC01093
RP11-789C1.1
LINC00890
ASS1P2
RP1-232P20.1
LINC00844
GBA3
HAND2-AS1
RP11-830F9.5
MT1JP
RP11-676J12.7
CTC-537E7.3
LINC00885
FENDRR
AC004540.4
LINC01018
MT2P1
MT1XP1
B
TPM
RP11-685F15.1
PCDH9-AS2
FAM99A
RP11-252E2.2
FAM99B
RP11-328K4.1
PLGLA
Adipose-Subcutaneous
Adipose-Visceral (Omentum)
Adrenal Gland
Artery-Aorta
Artery-Coronary
Artery-Tibial
Bladder
Brain-Amygdala
Brain-Anterior cingulate cortex (BA24)
Brain-Caudate (basal ganglia)
Brain-Cerebellar Hemisphere
Brain-Cerebellum
Brain-Cortex
Brain-Frontal Cortex (BA9)
Brain-Hippocampus
Brain-Hypothalamus
Brain-Nucleus accumbens (basal ganglia)
Brain-Putamen (basal ganglia)
Brain-Spinal cord (cervical c-1)
Brain-Substantia nigra
Breast-Mammary Tissue
Cells-Cultured fibroblasts
Cells-EBV-transformed lymphocytes
Cervix-Ectocervix
Cervix-Endocervix
Colon-Sigmoid
Colon-Transverse
Esophagus-Gastroesophageal Junction
Esophagus-Mucosa
Esophagus-Muscularis
Fallopian Tube
Heart-Atrial Appendage
Heart-Left Ventricle
Kidney-Cortex
Kidney-Medulla
Liver
Lung
Minor Salivary Gland
Muscle Skeletal
Nerve-Tibial
Ovary
Pancreas
Pituitary
Prostate
Skin-Not Sun Exposed (Suprapubic)
Skin-Sun Exposed (Lower leg)
Small Intestine-Terminal Ileum
Spleen
Stomach
Testis
Thyroid
Uterus
Vagina
Whole Blood
C
LUSC-FENDRR
LUSC-AC004540.4
LUSC-MT1JP
LUAD-FENDRR
LUAD-MT1JP
BRCA-HAND2-AS1
BRCA-MT1JP
Methylation (β-value)
1.0
0.5
0.0
NT
T
***
**
D
AC004540.4
mRNA expression
6
4
2
0
*
Cnt
DAC
H358

Figure S2. (A) List of the Top35 LNDH ordered as in Fig. 4A. In bold those lncRNAs that are preferentially expressed in the liver. **(B)** GTEx tissue-wide expression profiles of the liver specific lncRNAs (*RP11-685F15.1, PCDH9-AS2, FAM99A, RP11-252E2.2, FAM99B, RP11-328K4.1* and *PLGLA*). Visualization of the expression values (in TPM) of these lncRNAs across 54 tissues from GTEx. **(C)** DNA methylation levels (β-values) of the CpG found most significantly hypermethylated in the promoter region of the indicated lncRNAs in the patients from the LUSC, LUAD and BRCA TCGA cohorts for which both peritumoral (NT) and tumor (T) samples were available (LUSC n=41 pairs; LUAD n=29 pairs; BRCA n=90 pairs). The average is indicated as a line. **(D)** lncRNA *AC004540.4* expression by RT-qPCR in the lung cancer cell line H358 untreated or treated with 10 μM of 5-Aza-2′-deoxycytidine (DAC) for 7 days. *RPLP0* expression was used as housekeeping gene. The U the Mann-Whitney test was used for statistical analysis.* $p < 0.05$, ** $p < 0.01$, *** $p < 0.001$.

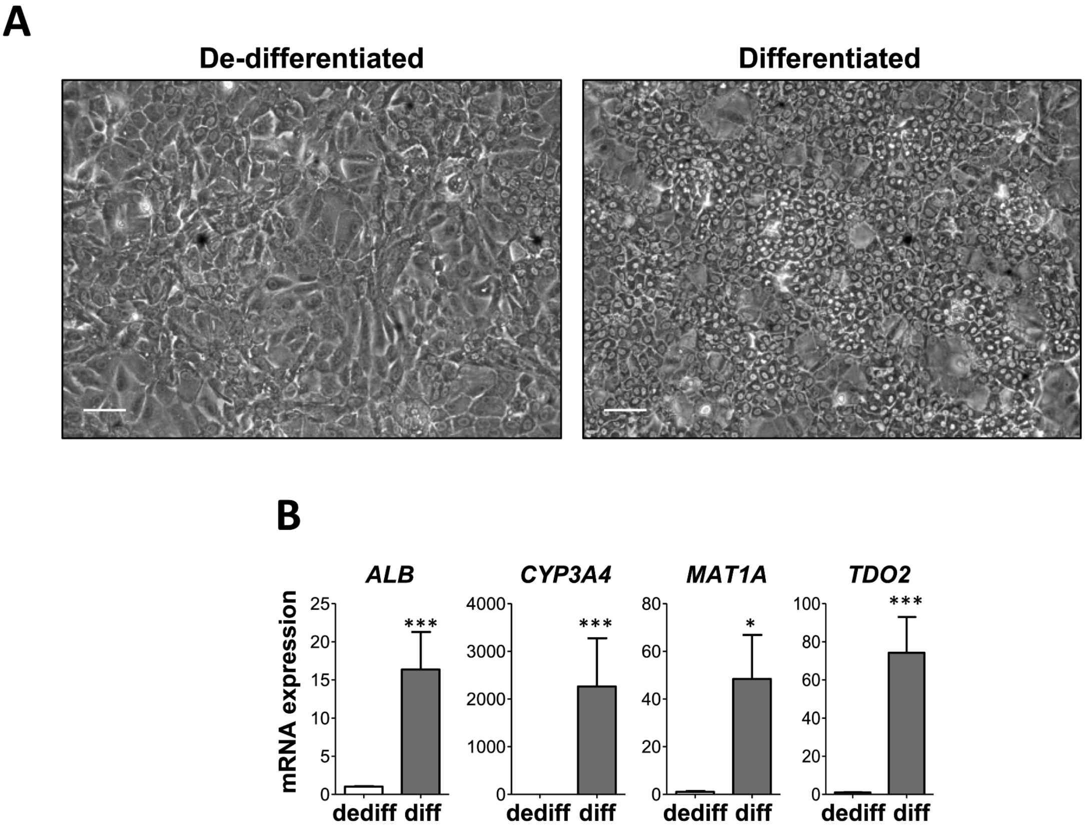

Figure S3. (A) Image of de-differentiated and differentiated HepaRG cells. Scale bar 20µm. **(B)** *ALB*, *CYP3A4* ,*MAT1A* and *TDO2* mRNA level by RT-q PCR in de-differentiated human hepatoma cell line HepaRG compared to well-differentiated HepaRG toward hepatocyte-like cells. *RPLP0* expression was used as housekeeping gene. The U the Mann-Whitney test was used for statistical analysis.* p < 0.05, *** p < 0.001.

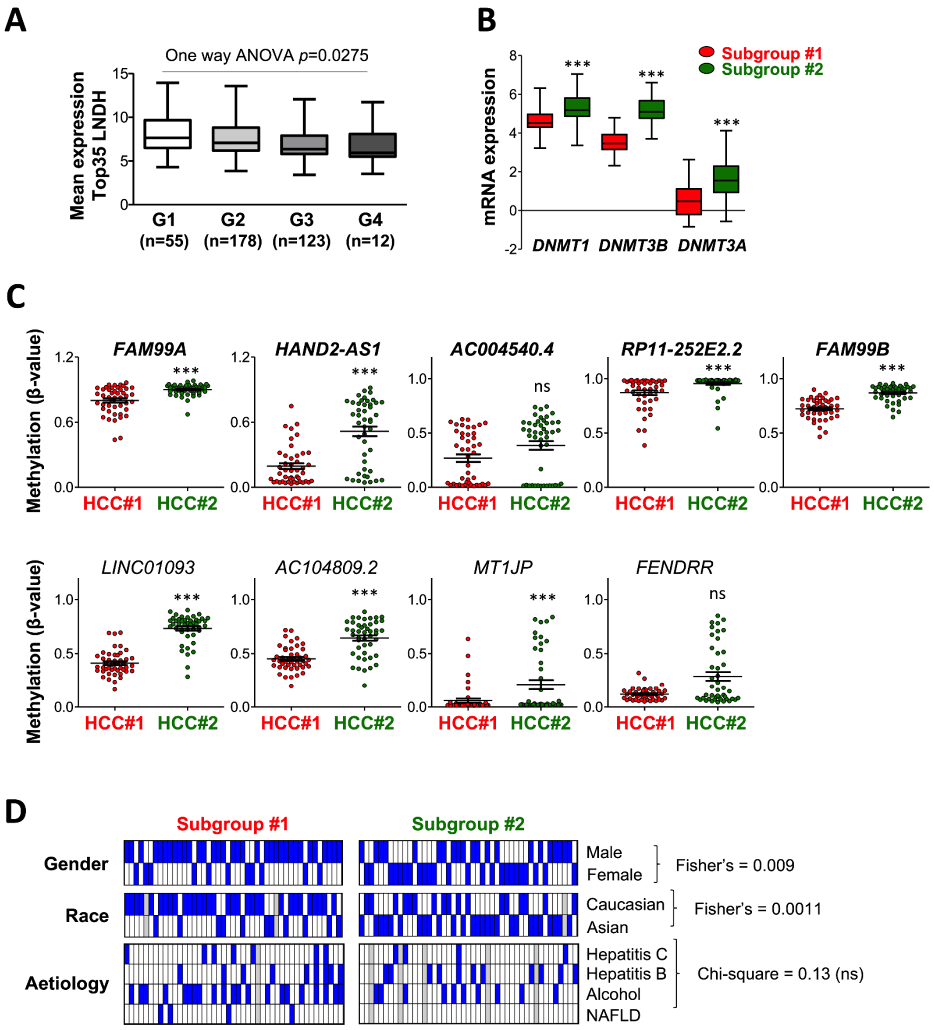

Figure S4. (A) Box plots reporting the mean expression of the Top35 LNDH in the TCGA HCC patients divided in four groups according to their histological grade (G1 to G4). **(B)** Box plots reporting the mRNA expression levels of *DNMT1*, *DNMT3B* and DNMT3A in the two established HCC subgroups from the LIHC TCGA cohort (Subgroup #1 in red and Subgroup #2 in green). **(C)** DNA methylation levels (β-value) of the CpG found most significantly hypermethylated in the promoter region of the indicated 9 lncRNAs in the two established HCC subgroups from the LIHC TCGA cohort (Subgroup #1 in red and Subgroup #2 in green). The average is indicated as a line. **(D)** Diagram reporting with a blue line the gender, race or presence of major HCC risk factors in the two Subgroups.

Statistical analysis is indicated for each parameter. The U the Mann-Whitney test was used for statistical analysis. *** $p < 0.001$, ns: nonsignificant differences.

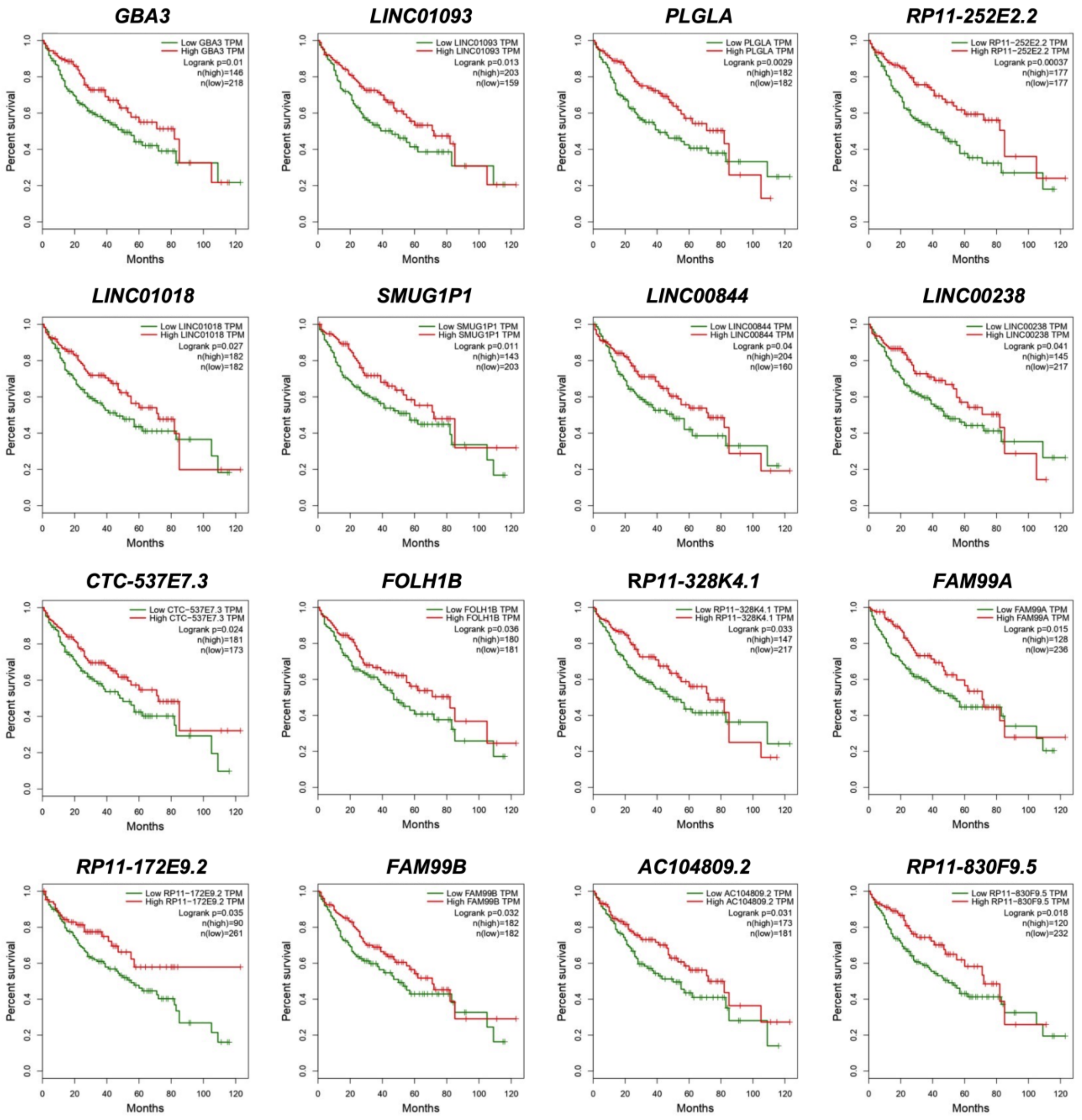

Figure S5. Kaplan-Meier curves reporting the overall survival of the HCC patients from the LIHC TCGA cohort divided in two groups according to the level of each indicated lncRNA. Graphs have been generated with GEPIA webpage (http://gepia.cancer-pku.cn/; accessed on 1 December 2021, and the log-rank test is indicated in each graph.
